# Genotoxic Effect of Dicyclopropanated 5-Vinyl-2-Norbornene

**DOI:** 10.3390/bios13010057

**Published:** 2022-12-29

**Authors:** Uliana S. Novoyatlova, Andrei G. Kessenikh, Olga V. Kononchuk, Sergey V. Bazhenov, Alexander A. Fomkin, Anna A. Kudryavtseva, Sergey V. Shorunov, Maxim V. Bermeshev, Ilya V. Manukhov

**Affiliations:** 1 Research Center for Molecular Mechanisms of Aging and Age-Related Diseases, Moscow Institute of Physics and Technology, 141701 Dolgoprudny, Russia; 2 Laboratory for Microbiology, BIOTECH University, 125080 Moscow, Russia; 3 Faculty of Physics, HSE University, 109028 Moscow, Russia; 4 Federal Research Center of Biological Systems and Agro-Technologies of RAS, 460000 Orenburg, Russia; 5 Topchiev Institute of Petrochemical Synthesis, RAS, 119071 Moscow, Russia

**Keywords:** *lux-*biosensor, genotoxicity, strained hydrocarbons, fuel

## Abstract

Dicyclopropanated 5-vinyl-2-norbornene (dcpVNB) is a strained polycyclic hydrocarbon compound with a high energy content, which makes it promising for the development of propellant components based on it. In this work, the genotoxic properties of dcpVNB were studied using whole-cell *lux*-biosensors based on *Escherichia coli* and *Bacillus subtilis*. It was shown that the addition of dcpVNB to bacterial cells leads to the appearance of DNA damage inducing the SOS response and Dps expression with slight activation of the OxyR-mediated response to oxidative stress. The highest toxic effect of dcpVNB is detected by the following *lux*-biosensors: *E. coli* pColD-lux, *E. coli* pDps, *B. subtilis* pNK-DinC, and *B. subtilis* pNK-MrgA, in which the genes of bacterial luciferases are transcriptionally fused to the corresponding promoters: P*_cda_*, P*_dps_*, P*_dinC_*, and P*_mrgA_*. It was shown that *lux*-biosensors based on *B. subtilis,* and *E. coli* are almost equally sensitive to dcpVNB, which indicates the same permeability to this compound of cell wall of Gram-positive and Gram-negative bacteria. The activation of P*_dps_* after dcpVNB addition maintains even in *oxyR* mutant *E. coli* strains, which means that the P*_dps_* induction is only partially determined by the OxyR/S regulon. Comparison of specific stress effects caused by dcpVNB and 2-ethyl(bicyclo[2.2.1]heptane) (EBH), characterized by the absence of cyclopropanated groups, shows that structural changes in hydrocarbons could significantly change the mode of toxicity.

## 1. Introduction

Strained cycloalkanes which have extra internal energy due to the deformation of valence bond angles are attractive for high-performance combustion applications, including the development of propellant components [1]. Based on the operating conditions of rocket technology, the development of rocket fuel components involves the selection of compounds that are highly efficient in terms of specific impulse energy but have low toxicity. Unsymmetrical dimethylhydrazine (UDMH) is a classic rocket propellant with a high calorific value; however, it is a highly toxic compound [2,3]. Due to these properties, the use of UDMH is associated with risks of environmental pollution during the operation of rocket technology, and especially in the cases of accidents at launches [4]. There are several studies showing the detection of UDMH in the environment and its impact on the ecosystems and human health [5,6,7]. Numerous studies have demonstrated the genotoxic effect of UDMH and its products of incomplete oxidation on the cell, which is determined by DNA alkylation [8,9,10] and DNA damage by reactive oxygen species (ROS) [11,12]. Strained hydrocarbon compounds based on cycloalkanes have two indisputable advantages: they are energetically more efficient than saturated hydrocarbons [13] and significantly less toxic than nitrogen-containing compounds like UDMH. It was shown in [14,15] that molecules consisting of one or two conjugated norbornanes: 2-ethyl(bicyclo[2.2.1]heptane) (EBH) and 2,2′-bis(bicyclo[2.2.1] heptane), respectively, do not possess the ability to alkylate DNA, but can induce the SOS response caused by cellular DNA damage by reactive oxygen species.

In this study, a strained polycyclic hydrocarbon compound dicyclopropanated 5-vinyl-2-norbornene (dcpVNB) was synthetized and its genotoxic characteristics were evaluated. dcpVNB is based on norbornane with addition of two cyclopropane groups and has an extra internal energy compared to norbornane due to deformation of valence bond angles. 

The dcpVNB toxicity evaluation was carried out using semi-specific whole-cell bacterial *lux*-biosensors. *Lux*-biosensors used for studying dcpVNB toxicity, were *E. coli* MG1655 and *B. subtilis* 168 cells transformed by set of hybrid plasmids in which bacterial luciferase genes (*lux*-genes) are transcriptionally fused to various stress promoters: P*_cda_*, P*_oxyS_*, P*_dps_*, P*_alkA_*, P*_dinC_*, or P*_mrgA_*. These *lux*-biosensors are semispecific and increase their luminescence in response to damage of cellular components correspondent for each biosensor [9,16,17,18,19]. These biosensors have a higher sensitivity compared to biosensors based on luminescence quenching. Taken together, it allows using these *lux*-biosensors to study the mechanisms of toxicity of various compounds and to evaluate toxic concentrations for cell. The *lux*-biosensor *E. coli* MG1655 pXen7 was used to evaluate the integral toxicity as it contains *lux*-genes *P. luminescens* ZM1 under control of its own constitutively transcribed promoter [20], in this case a decrease in the level of bioluminescence shows how compound affects vital functions of the cell.

## 2. Materials and Methods

### 2.1. Bacterial Strains and Plasmids

Bacterial strains and plasmids used in the study are listed in Table 1. 

*E. coli* MG1655 and MK2022 and *B. subtilis* 168 cells were used for obtaining *lux*-biosensors by transformation with corresponding plasmids.

All the plasmids used in the current work (presented in Table 1) were obtained from the National Base of Plasmids (https://nbp.biophystech.ru (accessed on 29 December 2022), BioPhysTech, Russia).

### 2.2. Culture Medium and Growth Conditions31

*E. coli* cells were cultured in liquid LB medium with continuous agitation at 200 rpm or on surface of agarized LB medium at 37 °C. The medium was supplemented with appropriate antibiotics (ampicillin 100 μg/mL and/or kanamycin 20 μg/mL).

For bioluminescence measurements overnight cultures of *E. coli* biosensor strains were diluted 1:100 in fresh LB supplemented with appropriate antibiotics and grown up to *OD_600_* = 0.1 − 0.2, the resulting cultures were used in experiments.

*B. subtilis* were cultured in liquid BHI medium with continuous agitation at 200 rpm or on surface of agarized BHI medium at 37 °C. Medium was supplemented with 50 mg/L tryptophan and 10 μg/mL chloramphenicol.

For bioluminescence measurements overnight cultures of *B. subtilis* biosensor strains were diluted 1:100 in fresh LB supplemented with appropriate antibiotics and grown up to *OD_600_* = 0.2 − 0.4, which corresponds to early logarithmic phase, the resulting cultures were used in experiments.

### 2.3. Measurement of Bioluminescence

Biosensor cells were sampled into the 200 μL subcultures in separate wells of 96-well plates, then 10 μL samples of tested compound (dcpVNB or control) were added. Cells were incubated without shaking at room temperature with regular measurements of total bioluminescence from each well. Bioluminescence was measured using SynergyHT (Biotek Instruments, Winooski, VT, USA). In general, *B. subtilis*–based biosensors are dimmer in comparison with *E. coli*-based ones, so more sensitive cuvette-reader Biotox-7BM (BioPhysTech, Russia) was used for validation of their luminescence measurements. Luminescence values were expressed in relative light units (RLU), specific to each luminometer.

### 2.4. Data Processing

Bioluminescence was measured several times during few hours. All experiments were conducted in three replications. Kinetic curves on graphs are the typical curves for the row of measurements (the kinetic curve which is consistent with the others). 

The maximum response amplitude for sample x is calculated due to following equation:

$$MRA\left(x\right)=max\left(\frac{L\left(t,x\right)}{L\left(t,ctrl\right)}\right),$$

where L(t,*x*)—the luminescence of probe *x* in the time point t, L(t,ctrl)—the luminescence of negative control, i.e., biosensor cells without addition of any toxicant at the time point t. 

For the determination of threshold concentrations, experiments with each biosensor were carried out with serial dilutions of dcpVNB. The threshold concentration is determined as the minimal concentration *x*, which gives noticeable induction (L(t, *x*) was higher than L(t, ctrl) in several time points by at least 2 values of measurement error). Further, threshold concentrations calculated from each experimental replicate were averaged and the standard deviation was calculated. 

### 2.5. P1 Transduction

P1 phage transduction of Δ*clpX:Kn* gene was performed according to [25]. Liquid P1 lysate was obtained using the donor strain *E. coli* BW25113 *ΔoxyR*, final phage stock contained approximately 5*108 pfu/mL. Overnight culture of the recipient strain *E. coli* MG1655 was mixed with 10 µL of liquid P1 phage lysate in 100 µL of P1 salt solution (10mM MgCl2, 5 mM CaCl2). The final bacterial-phage mix was spread on selective transduction plates containing 10 µm/mL kanamycin and 5 mM sodium citrate.

### 2.6. Chemicals

All chemicals were of analytical purity. Hydrogen peroxide was obtained from the firm “Ferraine” (Russia). Mitomycin C (MitC), methyl methanesulfonate (MMS), 5-vinyl-2-norbornene, acetic acid, methanol, diethyl ether, N-nitroso-N-methyl urea and other compounds were obtained from Sigma-Aldrich (St. Louis, MO, USA). For comparative measurements, EBH, synthetized earlier in study [14], was used. 

### 2.7. Synthesis of Dicyclopropanated 5-Vinyl-2-Norbornene (dcpVNB)

**Figure 1:**
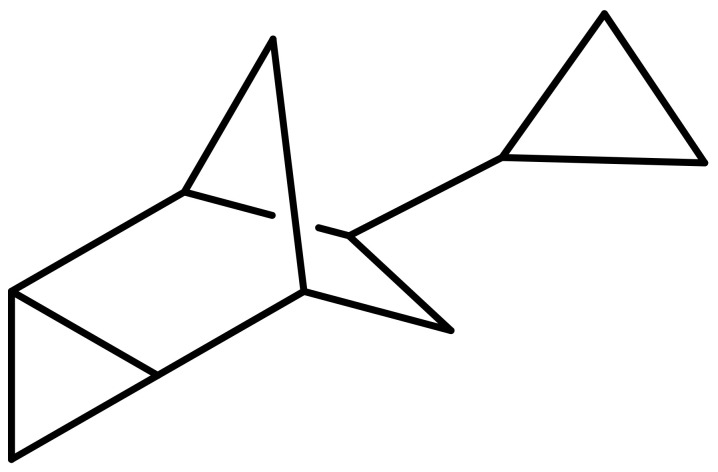
The molecular structure of dicyclopropanated 5-vinyl-2-norbornene (dcpVNB).

Dicyclopropanated 5-vinyl-2-norbornene (dcpVNB) was obtained according to the published methods [26,27,28,29,30]. The prepared dcpVNB consists of two types of strained cyclic moieties (a norbornane and two cyclopropane rings) and it contains eleven C and sixteen H atoms (Figure 1).

## 3. Results

### 3.1. DNA Damage: SOS Response and DNA Alkylation

The genotoxic effect of dcpVNB on bacterial cells was studied using *E. coli* MG1655 pColD-lux and *B. subtilis* 168 pNK-DinC *lux*-biosensors, which may detect the SOS response of bacterial cells. The *E. coli* MG1655 pAlkA-lux and *B. subtilis* 168 pNK-AlkA biosensors were used to detect the DNA alkylation caused by dcpVNB.

**Figure 2:**
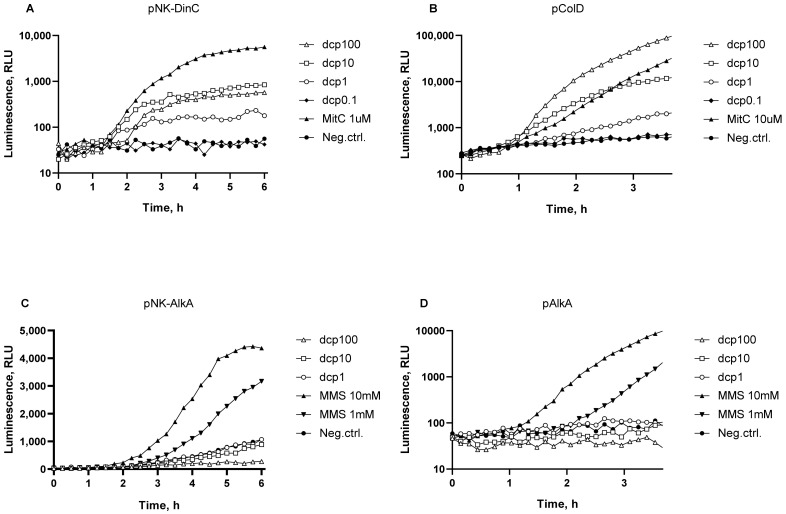
Typical kinetic curves of luminescence of *B. subtilis* 168 pNK-DinC (**A**), *E. coli* MG1655 pColD-lux (**B**), and *B. subtilis* 168 pNK-AlkA (**C**), *E. coli* MG1655 pAlkA-lux (**D**) cells after addition of dcpVNB. “Neg.ctrl.”—negative control, i.e., biosensor cells without addition of any toxicant; “MitC 1uM” and “MitC 10uM” – mitomycin C added to a final concentration of 1 μM and 10 μM, respectively; “MMS 10mM”, “MMS 1mM”—MMS added to final concentrations of 10 or 1 mM, respectively; “dcp100”, “dcp10”, “dcp1” and “dcp0.1”—dcpVNB added to final concentrations of 100, 10, 1, or 0.1 mg/mL, respectively.

Figure 2 shows the luminescence kinetic curves of biosensor cell cultures after addition of dcpVNB at final concentrations of 0.1–100 mg/mL. The antibiotic MitC was used as a positive control to activate P*_cda_* and P*_dinC_* SOS promoters (*E. coli* MG1655 pColD-lux and *B. subtilis* 168 pNK-DinC *lux*-biosensors, respectively). MitC produces DNA crosslinks, then the replication fork stops, single-stranded DNA sections are formed, and, as a result, the SOS response occurs. The alkylating agent MMS was used as a positive control to activate the P*_alkA_* promoter. 

As one can see from the Figure 2, the addition of dcpVNB at concentrations from 0.1% to 10% leads to DNA damage that results in the SOS response both in *E. coli* and *B. subtilis* cells (Figure 2A,B). The maximum possible response amplitude of *E. coli* pColD-lux biosensor is up to thousand [17]. In this experiment, the maximum response amplitude of the biosensor is about 200 during incubation with dcpVNB at concentration of 100 mg/mL. The threshold concentration of dcpVNB is about 0.5 mg/mL, approximately the same for both *E. coli* and *B. subtilis* SOS response specific biosensors.

In contrast to the observed SOS response, damage associated with DNA alkylation apparently does not occur as soon as promoter of DNA glycosylase P*_alka_* is not induced during cell culture incubation with dcpVNB (Figure 2C,D). A slight decrease in the luminescence of sensitive to alkylation biosensors with dcpVNB added at concentrations of 100 and 10 mg/mL (curves “dcp100” and “dcp10”) is associated with the general toxicity to bacterial cells.

### 3.2. Oxidative Stress

The most sensitive to oxidative stress *E. coli*-based *lux*-biosensor is *E. coli* MG1655 pOxyR-lux, which may detect an activation of the P*_oxyS_* promoter of the gene, encoding the regulatory RNA of OxyR/S regulon [9].

**Figure 3:**
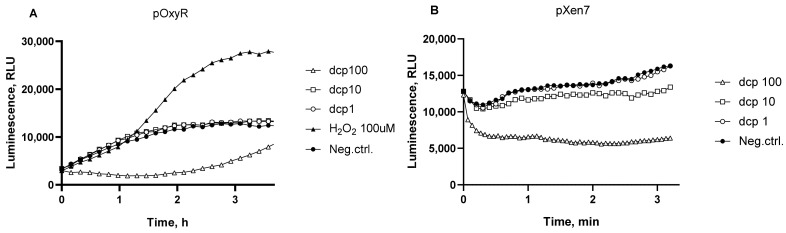
Typical kinetic curves of luminescence of *E. coli* MG1655 pOxyR-lux (**A**) and *E. coli* MG1655 pXen7 (**B**) cells after addition of dcpVNB. “Neg.ctrl.”—negative control, i.e., biosensor cells without addition of toxicant; “H_2_O_2_ 100uM”—hydrogen peroxide added to a final concentration of 100 μM ; “dcp100”, “dcp10”, and “dcp1”—dcpVNB added to a final concentration of 100, 10, and 1 mg/ml, respectively.

Figure 3A shows the luminescence kinetic curves of *E. coli* MG1655 pOxyR-lux biosensor strain after addition of dcpVNB at concentrations from 0.1 to 100 mg/mL. Hydrogen peroxide was used as a positive control to activate OxyR-regulated promoters. Cell cultures were incubated at room temperature without aeration for 4–6 h with periodic measurements of luminescence.

As can be seen from the data shown in Figure 3, the addition of dcpVNB to the cell culture causes a relatively weak oxidative stress, which is detected directly by the activation of the P*_oxyS_* promoter (Figure 3A). The luminescence is significantly reduced when the dcpVNB concentration is 100 mg/mL, it is apparently associated with the general toxicity to cells. The general toxicity was evaluated with the use of *E. coli* pXen7 biosensor with constitutive expression of luciferase (Figure 3B). A decrease in constitutive luminescence is observed at concentrations of 100 mg/mL and, to a lesser extent, at 10 mg/mL dcpVNB. On the Figure 3A a gradual rise in the “dcp100” curve can be observed after a fall in the first minutes of experiment, which indicates an activation of the P*_oxyS_* promoter, in contrast, there is no rise in luminescence of *E. coli* pXen7 biosensor in Figure 3B which means that its promoter is not induced.

**Figure 4:**
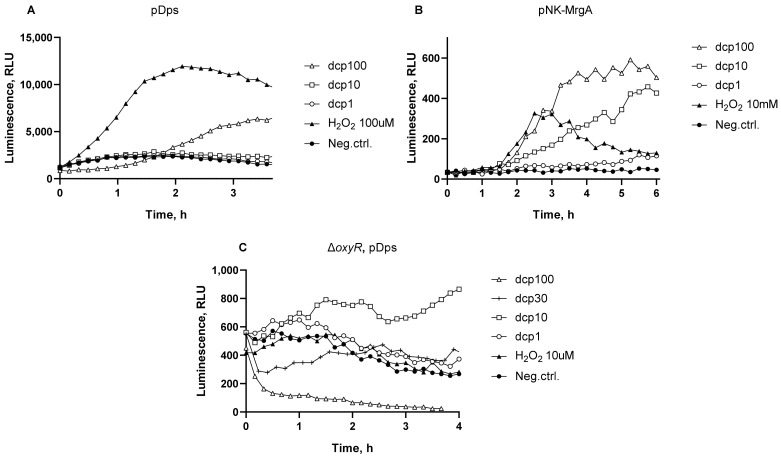
Typical kinetic curves of luminescence of *E. coli* MG1655 pDps (**A**), *B. subtilis* 168 pNK-MrgA (**B**), and *E. coli* MK2022 pDps (**C**) cells after addition of dcpVNB. “Neg.ctrl.”—control biosensor cells without addition of toxicant; “H_2_O_2_ 10uM”, “H_2_O_2_ 100uM” and “H_2_O_2_ 10mM”—hydrogen peroxide added to a final concentration of 10 µM, 100 µM and 10 mM, respectively; “dcp100”, “dcp30”, “dcp10”, and “dcp1”—dcpVNB added to a final concentration of 100, 30, 10, and 1 mg/mL, respectively.

Then, the ability of dcpVNB to induce oxidative stress in bacterial cells was investigated using *E. coli* MG1655 pDps and *B. subtilis* 168 pNK-MrgA. These biosensors show an activation of P*_dps_* and P*_mrgA_*, promoters regulating genes of DNA-binding ferritin-like proteins [31,32]. These genes are known to be regulated by several factors, including strong OxyR-mediated induction in response to oxidative stress [33,34,35]. Induction of the *E. coli* P*_dps_* and *B. subtilis* P*_mrgA_* stress promoters by addition of dcpVNB is illustrated in Figure 4. To test the possibility of OxyR-independent P*_dps_*activation, we studied the luminescence of the *E. coli* MK2022 strain with a deletion of the *oxyR* regulatory gene (Figure 4C).

The *E. coli* P*_dps_* promoter and *B. subtilis* P*_mrgA_* have a relatively high response amplitude (Figure 4A,B) and a threshold concentration of dcpVNB for these biosensors is approximately 1 mg/mL. Figure 4C shows that a certain activation of the P*_dps_* promoter also occurs in the Δ*oxyR* mutant strain. When dcpVNB was added to MK2022 cells, activation was noticeable at concentrations from 1 to 10 mg/mL. dcpVNB at concentration of 100 mg/mL for Δ*oxyR* strain is more toxic than for the wild type and luminescence of cells is significantly reduced. These results indicate the ability of dcpVNB to activate promoters of DNA-binding ferritin genes in an OxyR-independent manner.

### 3.3. Comparison of SOS Response and Oxidative Stress in Cells from dcpVNB and EBH

**Figure 5:**
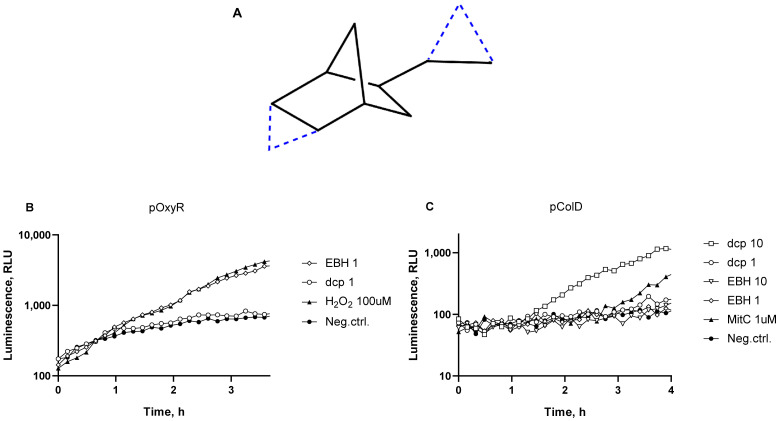
(**A**)—Molecular structure of EBH (black solid lines). The difference in structure of dcpVNB and EBH (additional two carbons and four bonds) is given in blue dashed lines. (**B**,**C**)—Typical kinetic curves of luminescence of *E. coli* MG1655 pOxyR-lux (**B**) and *E. coli* MG1655 pColD-lux (**C**) cells after addition of dcpVNB and EBH. “Neg.ctrl.”—negative control, biosensor cells without addition of toxicant; “H_2_O_2_ 100uM”—hydrogen peroxide added to a final concentration of 100 µM; “MitC 1uM”—mitomycin C added to a final concentration of 1 µM; “dcp10” and “dcp1”—dcpVNB added; “EBH 1” and “EBH 10”—EBH added to final concentrations of 10 and 1 mg/mL, respectively.

In our previous work, the toxicity of EBH, a compound with a structure quite similar to dcpVNB but without cyclopropane groups (Figure 5A), was investigated [14]. We noted that dcpVNB produce very little amount of oxidative stress and strongly induce the SOS response, while EBH showed presumably oxidative damage of cells. To investigate this difference, we compared the genotoxic effects of dcpVNB and EBH in one experiment with the use of the following biosensors: *E. coli* MG1655 pOxyR-lux and *E. coli* MG1655 pColD-lux.

Compounds dcpVNB and EBH were added to biosensor cells in various concentrations, and then the samples were incubated at room temperature without aeration for 3–4 h with periodic measurements of bioluminescence (Figure 5).

As can be seen from Figure 5, EBH possesses a high ability to activate the oxidative stress in the cells, while dcpVNB possesses a low one (Figure 5B), and vice versa, while the SOS response is activated in the cells, dcpVNB has high efficiency to induce SOS response, while the EBH ability to damage DNA is relatively low (Figure 5C).

Collected data was processed and the values for threshold concentrations were summarized in the Table 2.

## 4. Discussion

Incomplete oxidation of most hydrocarbon compounds leads to the appearance of various reactive oxygen species in the aquatic environment, in particular alkyl hydroperoxides, alkyl radicals, and other compounds capable of causing oxidative stress in both pro- and eukaryotic cells [37,38]. Compounds with strained bonds, as shown in [1,13], are oxidized mainly by the radical mechanism. The radical mechanism of oxidation along with the increased reaction energy suggests that the genotoxic effect of strained compounds should be determined mainly by reactions of reactive oxygen species formation [15]. For the EBH compound, these statements are generally confirmed by experiments on *E. coli* [14], demonstrating the activation of the OxyR/S regulon. In contrast to EBH, the addition of dcpVNB to the biosensor cell culture weakly activates the P*_oxyS_* promoter, which means almost no oxidative damage occurs. Moreover, the data presented in Figure 5 demonstrate that there is a fundamental difference in the mechanisms of genotoxicity of EBH and dcpVNB compounds. EBH is characterized by the formation of ROS, which cause DNA damage, that is consistent with the data [10], while dcpVNB has a ROS-independent SOS response, characterized by a high response amplitude of P*_cda_*, though the oxidative stress at the same concentrations of dcpVNB is absent. The ROS-independent toxic effect of dcpVNB on bacterial cells is supported by data demonstrating the activation of *E. coli* P*_dps_* and *B. subtilis* P*_mrgA_*ferritin promoters. This result is somewhat unexpected, since it was assumed that the key contribution to the activation of P*_dps_* and P*_mrgA_* is brought by OxyR/S. The activation of the P*_dps_*promoter in the Δ*oxyR* strain explains this phenomenon by the participation of other regulators. It is known that, in addition to OxyR/S, these ferritin promoters can be activated by several other regulators, in particular, CsrA, FIS, H-NS, σ^38^, and by the sporulation signal in bacilli [33,34,37,38]. It appears that the ROS-independent toxic effect of dcpVNB induces the activation of some of these regulators.

Thus, compared to EBH, the dicyclopranated form of norbornane, dcpVNB induces a stronger SOS response and less severe oxidative damage to the cell. This is a key difference in the mechanisms of toxicity of two strained cycloalkanes differing only in two cyclopropanated groups. Specific targets in the cell for the molecule of dcpVNB still require further research.

The data in Table 2, comparing the toxicological properties of strained hydrocarbon compounds based on cycloalkanes, shows that the substance dcpVNB studied in this article is averagely 1000 times less toxic than UDMH in terms of its ability to cause oxidative stress in cells and DNA damage that stops the replication fork (SOS response). According to the mechanism of genotoxicity, the disability of dcpVNB to methylate DNA sharply reduces its carcinogenic properties compared to UDMH.

## 5. Conclusions

In conclusion, it should be noted that, in general, the toxicity of strained cyclic compounds is significantly lower than that of unsymmetrical dimethylhydrazine, which is currently used as the most energy-efficient propellant component. 

In this study, we have shown the genotoxic properties of a new compound that has the potential for widespread use as a highly efficient hydrocarbon-based fuel. It is important to note that dcpVNB has no alkylating effect, which means that it has no mutagenic properties, as UDMH has. The oxidizing effect on cell components and DNA damage coupled with formation of single-stranded DNA are not so dangerous for multicellular organisms and do not have a long-lasting effect. Threshold concentrations of strained cycloalkanes causing oxidative stress are much lower than of UDMH, so it makes them less toxic. The use of dcpVNB may provide opportunities for making rocket industry much safer for environment and human health. 

We demonstrated here that changes in the structure of the strained cycloalkane caused a sharp change in the mechanism of toxicity. In particular, due to the appearance of two cyclopropane groups in the structure, the ability of the compound to cause oxidative damage to cells was reduced and, at the same time, the ability to damage DNA was increased. 

## Figures and Tables

**Table 1:** Bacterial strains and plasmids used in the study.

Name	Description	Source	
Strains		
*E. coli* MG1655	*F- ilvG rfb-50 rph 1*	(VKPM, Russia)
*E. coli* BW25113 Δ*oxyR*	*Δ(araD-araB)567 ΔlacZ4787(::rrnB-3) λ- rph-1 Δ(rhaD-rhaB)568 hsdR514 ΔoxyR::Km* *^r^*	[21]
*E. coli* MK2022	*ΔoxyR::Km^r^* (obtained by transfer of gene deletion *ΔoxyR* from BW25113 Δ*oxyR* to MG1655 using P1 transduction)	This study
*B. subtilis* 168	*trpC2*	(VKPM, Russia)
Plasmids		
pNK-AlkA	pPL_ABCDExen vector [22] with insertion of the *B. subtilis* P*_alkA_* promoter; P*_alkA_* is transcriptionally fused with *luxCDABE P. luminescens*. Trimethoprim (Tp^r^), chloramphenicol (Cm^r^), and ampicillin (Ap^r^) resistance.	[16]
pNK-DinC	As pNK-AlkA, but *B. subtilis* P*_dinC_* promoter is used. Tp^r^, Cm^r^, Ap^r^	[16]
pNK-MrgA	As pNK-AlkA, but *B. subtilis* P*_mrgA_* promoter is used. Tp^r^, Cm^r^, Ap^r^	[16]
pAlkA-lux	pDEW201 [23] vector with insertion of *E. coli* P*_alkA_* promoter transcriptionally fused with *luxCDABE P. luminescens*. Ap^r^	[8]
pDps	As pAlkA-lux, but *E. coli* P*_dps_* promoter is used. Ap^r^	[24]
pOxyR-lux	As pAlkA-lux, but *E. coli* P*_oxyS_* promoter with the gene *oxyR* is used. Ap^r^	[17]
pColD-lux	As pAlkA-lux, but P*_cda_* from plasmid ColD-CA23 is used. Ap^r^	[17]
pXen7	pUC18-based plasmid constitutively expressing *luxCDABE* genes. Ap^r^	[20]

**Table 2:** Threshold concentrations for dcpVNB, EBH, and UDMH detectable with *lux*-biosensors, used in this study. * Concentrations were obtained earlier [10,36]. ** ”nd”—not determined. ***“ne”- not evaluated.

Biosensors	dcpVNB, mM	EBH, mM	UDMH *, mM	Note
*E. coli* pAlkA-lux	nd **	nd	2*10^−2^	Alkylation of DNA
*B. subtilis* pNK-AlkA	nd	ne ***	ne	
*E. coli* pColD-lux	2.3 ± 0.7	5.6 ± 1.9	8*10^−3^	DNA damage, leading to SOS response
*B. subtilis* pNK-DinC	3.5 ± 1.2	ne	ne	
*E. coli* pDpS	54 ± 8	ne	ne	Oxidative stress
*B. subtilis* pNK-MrgA	6.0 ± 2.1	ne	ne	
*E. coli* pOxyR-lux	470 ± 50	4.2 ± 1.8	3*10^−3^	Oxidation by hydrogen peroxide
*E. coli* pXen7	67 ± 15	43 ± 7	2	Total toxicity, decrease in luminescence correlates with the number of living cells

## Data Availability

The data is contained within the article.
